# Milk Bank Pooling Practices Impact Concentrations and Variability of Bioactive Components of Donor Human Milk

**DOI:** 10.3389/fnut.2020.579115

**Published:** 2020-10-06

**Authors:** Bridget E. Young, Katherine Murphy, Laraine L. Borman, Rebecca Heinrich, Nancy F. Krebs

**Affiliations:** ^1^Department of Pediatrics Allergy and Immunology, University of Rochester School of Medicine and Dentistry, Rochester, NY, United States; ^2^Mothers' Milk Bank, Rocky Mountain Children's Health Foundation, Arvada, CO, United States; ^3^Department of Pediatrics - Section of Nutrition, University of Colorado School of Medicine, Aurora, CO, United States

**Keywords:** donor human milk, insulin, IgA, milk bank, breastfeeding

## Abstract

**Background:** Donor human milk (DHM) bank practices, such as pasteurization and pooling according to postpartum age of milk donations and number of donors included in a pool may impact the resulting concentration of bioactive components of DHM.

**Aims:** We determined the impact of Holder pasteurization, postpartum milk age, and pool donor number (number of donors included in a pool) on resulting concentrations of total immunoglobulin A (IgA; which provides immune protection to the recipient infant) and insulin (an important hormone for gut maturation).We also documented inter-relationships between these bioactive components and macronutrients in DHM pools.

**Methods:** Pre and post-pasteurization aliquots of 128 DHM samples were obtained from the Rocky Mountain Children's Foundation Mother's Milk Bank (a member of the Human Milk Banking Association of North America, HMBANA). Macronutrients were measured via mid-infrared spectroscopy. Total IgA was measured via customized immunoassay in skim milk and insulin was measured via chemiluminescent immunoassay.

**Results:** Mean post-pasteurization total IgA concentration was 0.23 ± 0.10 (range: 0.04–0.65) mg/mL a 17.9% decrease due to pasteurization (*n* = 126). Mean post-pasteurization DHM insulin concentration was 7.0 ± 4.6 (range: 3–40) μU/mL, a decrease of 13.6% due to pasteurization (*n* = 128). The average DHM pool postpartum milk age was not associated with total IgA or insulin concentrations, but pool donor number was associated with bioactive components. Pools with only one donor had lower total IgA and lower insulin concentrations than pools with at least 2 donors (*p* < 0.05). Increasing the number of donors in a pool decreased the variability in total IgA and insulin concentrations (*p* < 0.04).

**Conclusion:** Increasing the number of donors included in DHM pools may help optimize bioactive components in DHM received by premature infants. These results help inform milk banking practices to decrease compositional variability in produced DHM pools.

## Introduction

The American Academy of Pediatrics recommends pasteurized donor human milk (DHM) as the best option to feed a premature infant when mother's own milk is unavailable (1). The Human Milk Banking Association of North America (HMBANA) supplies millions of ounces of pasteurized DHM annually to recipient infants (2). The majority of this milk is provided to premature infants in the Neonatal Intensive Care Units (NICU), due to the protective effects of DHM against necrotizing enterocolitis (3, 4).

Human milk composition is dynamic, changing over the course of a feed, over the course of a day, and over the course of lactation. For these reasons, DHM banks routinely pool milk donations from multiple donors in order to limit extreme variation. Criteria used to select individual donations for pooling are not standardized and vary among milk banks. Currently, milk postpartum age (age of the infant at the time milk was expressed) is not typically taken into account when pooling individual donations. Furthermore, milk banks each have different acceptance criteria for milk age.

Upon pooling, DHM is aliquoted and pasteurized using Holder Pasteurization (62.5°C for 30 min and rapid cooling). At this stage, bottles are frozen and distributed. It is well-documented that Holder Pasteurization reduces the concentration of various bioactive components of HM. However, the degree to which individual bioactive components are degraded is widely variable, and not consistently reported in the literature (5).

A biologically active component of HM is immunoglobulin A (**IgA**) (6). HM total IgA is one of the components that provides breastfed infants passive protection against infections during infancy (7, 8). Secretory IgA (a dimer of two IgA attached to a secretory component) is the predominant type of immunoglobulin A present in HM and provides infants with passive immune protection (6, 7). Total IgA concentrations in HM decrease over the first year of lactation (6, 9). Additionally, Holder Pasteurization has been reported to reduce IgA concentrations by 20–62% (5, 9, 10).

HM insulin plays important roles in infant intestinal maturation (11), may improve feed tolerance (11), impact the developing microbiome (12), and has been linked to infant growth patterns and body fat accrual (13). Insulin in HM is predominantly impacted by maternal insulin sensitivity (14), a factor not considered in the donor milk pooling process. Additionally, current data suggest that HM insulin decreases over the first month of lactation (14), and is also significantly decreased by Holder Pasteurization between 13–46% (15, 16).

Because IgA represents a significant component of total HM protein, and because both HM insulin and total fat have been reported to correlate with maternal BMI (17–19), we also tested for correlations between DHM IgA and insulin with DHM macronutrients.

Given the importance of these bioactive components of HM, we aimed to characterize the mean and variability of total IgA and insulin concentrations in HMBANA-produced DHM pools and investigate the impact of Holder Pasteurization and relationships between milk postpartum age and number of donors included in a pool.

## Materials and Methods

### Donor Human Milk Bank Samples

This research received designation as “Not Human Subject Research” by the Colorado Multiple Institution Review Board.

Pre- and post-pasteurization samples from 128 DHM pools were obtained from the Colorado Mothers' Milk Bank (Rocky Mountain Children's Health Foundation, Arvada, CO). This milk bank selects individual donations to pool based on the expiration date of donated milk. Milk donations are considered to “expire” 12 months post expression date. DHM pools are then aliquoted, pasteurized in glass bottles via Holder Pasteurization and frozen for distribution.

Preterm milk pools included only human milk expressed by a mother who delivered at or before 36 weeks gestation, within the first 4 weeks postpartum and up until her infant's corrected age is 40 weeks. Pool donor number was defined as the number of milk donors represented in each pool. Pool postpartum age was defined as the unweighted mean of the postpartum ages of all the donations included in any given pool as previously described (17).

### Milk Analyses—Total IgA

Total IgA concentrations of pre- and post-pasteurization samples were measured using an adapted version of a commercially available immunoassay (Bethyl Laboratories, Montgomery, Texas). A flat bottom 96 well Maxisorp plate was coated with a 1:100 dilution of anti-human IgA (Bethyl Lab A80–102A) in 0.05M carbonate-bicarbonate: 100 uL per well, overnight at 4°C. The following day, 200 uL of 1% bovine serum albumin (BSA) in PBS was added to each well to block the plate for 30 min at room temperature. Skim milk was generated by spinning samples at 10,000 g for 10 min at 4°C. Skim milk samples were diluted 1:5000 in 1% BSA in PBS and then incubated on the plate for 2 h at room temperature. Assay controls were prepared from a serial dilution of control serum (Bethyl Lab RS10–110) in 1% BSA in PBS. Secondary antibody (Bethyl Lab A80–102P) was added at a concentration of 1:100,000 in 1% BSA in PBS and incubated for 1 h at room temperature: 100 uL per well. Following the 1 h incubation time, 50 uL of stop solution (2M H_2_SO_4_) was added to each well.

The plate was washed after the following: blocking, sample incubation, and secondary antibody incubation using a BioTek Microplate Washer and a PBS-T solution with 0.05% Tween-20 for five cycles of a 300 μL wash. The absorbance was then read at 450 nm and a 4PL standard curve was plotted within 30 min of adding the stop solution. Samples were assayed in duplicate and re-assayed if %CV of duplicates was >25%. The assay average %CV between replicates was <10%.

### Milk Analyses—Macronutrients and Insulin

DHM macronutrients (fat, protein, carbohydrate, and caloric density) were formerly assessed using the Miris Human Milk Analyzer (Miris AB, Uppsala Sweden) as previously reported (17). Insulin concentrations were measured in pre- and post-pasteurization skim milk samples via chemiluminescent immunoassay utilizing an automated immunoassay platform (Beckman Coulter), as previously reported (14).

### Statistical Analyses and Calculations

Comparison of milk composition between Preterm vs. Term DHM pools and by pool donor number was conducted using non-parametric tests. For comparison by pool donor number, DHM pools were categorized as consisting of donations from 1, 2, or ≥3 donors. A comparison of milk composition between pre-pasteurized and post-pasteurized samples was conducted using paired *t*-tests.

Simple linear regression was used to determine if the pool Postpartum Age was related to milk composition, and if any individual milk components were inter-correlated. Log transformation was used on any non-linear variables. The normality of these regression's residuals was established to ensure model assumptions were satisfied. The Brown–Forsythe test was performed to test whether variation in log DHM Total IgA and Insulin concentrations differed by pool donor number. Analyses were performed using JMP Pro 14 (SAS, Cary NC).

## Results

### Characteristics of Pools

Out of the 128 DHM pools, 5 (3.9%) of the pools were “Preterm,” and 4 (3.1%) of the pools were “Dairy Free” meaning donors completely omitted dairy products from their diet. Data regarding pool donor number (number of donors in each pool), pool volume, and milk age have been previously reported (17). Of the 128 pools, 55 pools consisted of 1 donor; 62 pools consisted of 2 donors; and 11 pools consisted of ≥3 donors.

### Pool Composition–Total IgA

Two of the 128 post-pasteurization samples were inconclusive for total IgA analysis due to high %CV, giving a final sample size for post-pasteurization total IgA analysis of 126. Two of the term post-pasteurization samples were not included in the final data analysis in order to not bias results, as both were outliers over five standard deviations from the IgA mean (1.575 and 1.212 mg/mL). Excluding these data points did not change the nature of reported relationships.

As expected, given the Holder pasteurization process, total IgA concentrations were higher in pre-pasteurized DHM pools. The mean ± SD of total IgA concentrations in pre-pasteurization samples was 0.31 ± 0.14 mg/mL (*n* = 128) vs. 0.23 ± 0.09 mg/mL in post-pasteurization samples (*n* = 126). The total IgA concentrations in post-pasteurization samples decreased on average 0.08 mg/mL. The range in decrease was −0.61 to + 0.19 mg/mL), or 17.9% (*p* < 0.001; Table 1).

**Table 1:** Total IgA and Insulin Concentrations in Pasteurized Donor Human Milk.

**Component**	**Sample**	**Mean**	**Standard deviation**	**Range**	**Percent decrease**
Total IgA (mg/mL)	Pre-pasteurization	0.31	0.14	0.06–0.84	17.9
	Post-pasteurization^a^	0.23	0.10	0.04–0.62	
Insulin (μU/mL)	Pre-pasteurization	8.1	5.7	3.0–54.0	12.6
	Post-pasteurization	7.0	4.6	3.0–40.0	

a *Sample size = 126; Otherwise, sample size = 128*.

**Figure 1:**
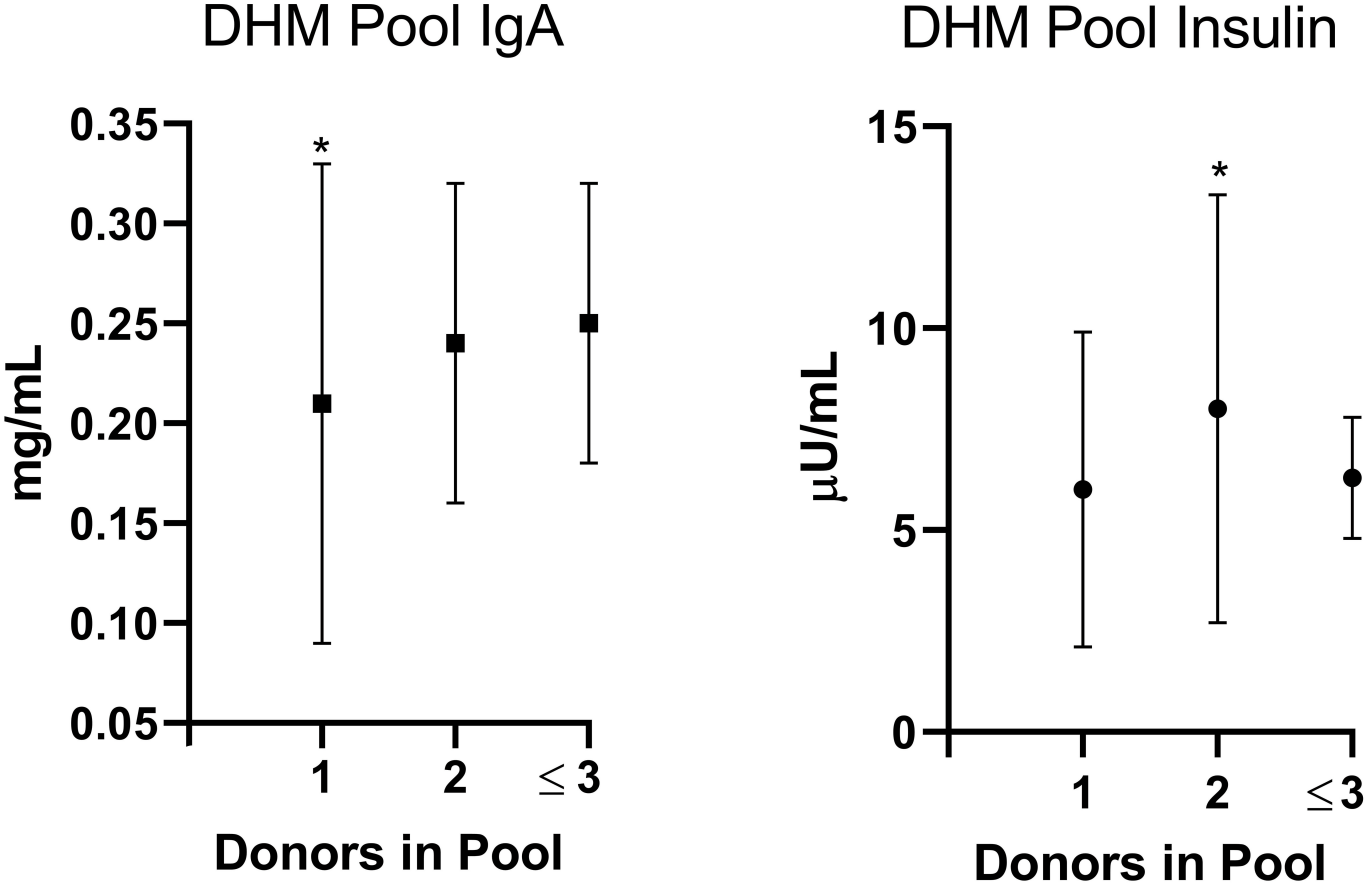
Donor Human Milk (DHM) Immunoglobulin A (IgA, mg/mL) and Insulin (μU/mL) concentrations differ by Pool donor number (the number of donors included in the pool). Pools including one (*n* = 54) vs. two (*n* = 61) vs. ≤3 (*n* = 11) donors have different concentrations of IgA (*p = 0.04) and Insulin (*p = 0.003), by Wilcoxan test. Furthermore, the variation in concentrations was lowest in DHM pools consisting of ≤3 donors (IgA *p* = 0.027; Insulin *p* = 0.034). Mean ± standard deviation presented.

The DHM total post-pasteurization IgA concentrations were not correlated with pool postpartum milk age. DHM total post-pasteurization IgA concentrations did differ by pool donor number: DHM pools with one donor had a lower total IgA concentration than pools with at least two donors (*p* = 0.04; Figure 1). Increasing the number of donors in a pool significantly decreased the variability in resulting total IgA concentrations (*p* = 0.027).

The DHM total post-pasteurization IgA concentrations did not significantly differ between Preterm and Term pools, nor was DHM Total post-pasteurization IgA associated with DHM macronutrient composition.

### Pool Composition—Insulin

DHM insulin decreased an average of 12.6% following pasteurization; from 8.1 ± 5.7 μU/mL (range: 3–54 μU/mL) to 7.0 ± 4.6 μU/mL (range: 3–40 μU/mL). The range in decrease was −15 to + 3 μU/mL. DHM post-pasteurization insulin concentrations were not associated with pool milk age.

DHM total post-pasteurization insulin concentrations did differ by pool donor number: DHM pools with one donor had lower insulin concentrations than pools with 2 donors (*p* = 0.003; Figure 1). Increasing the number of donors in a pool significantly decreased the variability in resulting total insulin concentrations (*p* = 0.034).

DHM insulin concentrations did not differ between term and preterm pools. The log of DHM pool insulin concentrations was positively associated with DHM fat content (*p* < 0.0001, *R*^2^ = 0.16, *n* = 128), and caloric density (*p* < 0.0001, *R*^2^ = 0.15, *n* = 128).

## Discussion

This study demonstrates a large variability in DHM total IgA and insulin concentrations. Holder pasteurization resulted in significant decreases in both bioactive components. While postpartum milk age was not associated with variation in total IgA and insulin concentrations, pool donor number was associated with significant differences in the mean and total variation of both.

This data corroborates others' findings suggesting that total IgA is affected by the pasteurization process. Other studies reported a decrease in total IgA due to Holder Pasteurization within the range of 20–62% (5, 9, 10), which is higher than we detect here (18%). Previously reported studies simulated milk pooling in a laboratory setting in smaller sample sizes, whereas this study includes a larger sample size and DHM directly from a HMBANA milk bank, which may account for the difference in percent–decrease.

Even though milk IgA decreases over time in the first year of lactation, DHM total IgA was not associated with milk postpartum age. This is in contrast to our previous work showing that zinc concentrations, which also decline over the course of lactation, are inversely correlated with DHM pool postpartum age (17). These zinc data are similar to others' work showing that DHM amino and fatty acid composition is also related to postpartum age (20). However, other studies of macronutrient composition of DHM pools have documented that subject effect (i.e., variability between donors) had a higher impact on macronutrient composition than time effect (i.e., pool postpartum age) after 4 weeks postpartum (21, 22), even though human milk macronutrient composition is known to change over time. These findings reflect our own with total IgA concentrations.

DHM total IgA in postpartum samples did differ by pool donor number with lower concentrations in pool from one donor, and less variability in pools with 3+ donors. This finding validates the HMBANA policy of pooling milk, ideally from three to five donors (23). However, milk banks may occasionally face limited capacity to meet this recommendation based on fluctuations in availability of donations. In a study of over 300 DHM pool samples from 20 milk banks, 55% of studied pools consisted of donations from ≥3 donors (24). This study collected 15 random samples from 20 milk banks, whereas our data represent consecutive DHM pools generated over several different days in one bank which likely contributes to the differences in the amount of DHM pools consisting of ≥3 donors. Given that IgA in DHM may protect infants from infection, any efforts to optimize and standardize its provision to medically fragile infants is worthwhile.

Our data suggest that a recipient infant could be exposed to a wide range in insulin concentrations from one DHM pool to another. The role of insulin in HM is complex and yet to be fully characterized (25). Animal data suggest that milk insulin directly affects intestinal gene expression and maturation (5, 10, 26–29). Data from human infants indicate that milk insulin also contributes to intestinal lactase expression and feed tolerance (30), as well as playing a role in regulation of infant weight gain (31) and body composition (13).

It is noteworthy that we detected an average decrease of 13% in insulin due to Holder pasteurization, with 34% of samples showing no decrease. This reduction is less than the 46% decrease in average insulin reported by Ley et al. (15), but very similar to the 13% reduction in averages reported by Vass et al. (16). Similar to total IgA, DHM insulin was not related to milk pool age, but was related to pool donor number with lower concentrations in pools from one donor, and lower variation in pools with ≥3 donors.

We also detected a correlation between DHM insulin and total fat concentrations (and thus caloric density as well). This relationship may reflect the underlying relationship between maternal BMI and both human milk insulin concentrations (17), and milk fat, as several studies have reported that women with higher BMI and/or obesity produce milk with higher fat content (18, 19). Currently, maternal phenotype, including BMI, is not taken into account during the pooling process. Given the shortage of milk donations in the US, it does not seem warranted that maternal BMI be considered during pooling.

A strength of our study is usage of pre- and post-pasteurized DHM pools produced by a HMBANA milk bank, as opposed to small batch lab-generated pools. An additional strength of the study is the large sample size, which allows for an accurate estimate of the variation in pool characteristics. Our inability to link DHM pools and the total IgA and insulin concentrations to outcomes of recipient infants, or to characteristics of individual donors may be considered a weakness. Furthermore, these samples were generated from one milk bank where 9% of pools consisted of ≥3 donors over the days of pool production studied, which may not be representative of other milk banks. These findings help inform milk bank practices to optimize the benefit of DHM provided to recipient infants. Additionally, these findings help inform neonatologists about the mean and variability of bioactive components in DHM fed to premature infants.

In conclusion, we have shown that DHM pools yield a wide range in concentrations of two potent bioactive components: total IgA and insulin. The number of donors included in a DHM pool affect both the final concentrations and the variability in total IgA and insulin. As such, there may be a benefit to standardizing the procedures for pool generation across independent milk banks to provide a more uniform “dose” of these bioactives to recipient premature infants. Lastly, these data support the need for both social and cultural initiatives to ensure donor milk banks have enough donation availability to meet pooling goals.

## Data Availability Statement

The raw data supporting the conclusions of this article will be made available by the authors, without undue reservation.

## Author Contributions

BY, LB, RH, and NK contributed to the conception and design of the study. LB, RH, BY, and KM collected the data. BY organized the database. BY and KM performed the statistical analysis. BY and KM wrote the first draft of the manuscript. All authors contributed to manuscript revisions, read, and approved the submitted version.

## Conflict of Interest

The authors declare that the research was conducted in the absence of any commercial or financial relationships that could be construed as a potential conflict of interest.
